# Recommendations to enhance constructivist-based learning in Interprofessional Education using video-based self-assessment

**DOI:** 10.3205/zma001032

**Published:** 2016-04-29

**Authors:** Uta Dahmen, Christine Schulze, Claudia Schindler, Katharina Wick, Dominique Schwartze, Andrea Veit, Ulrich Smolenski

**Affiliations:** 1 University Hospital Jena, Department of General, Visceral, and Vascular Surgery, Experimental Transplantation Surgery, Jena, Germany; 2 University Hospital Jena, Institute of Psychosocial Medicine and Psychotherapy, Jena, Germany; 3 Vocational School of Health and Social Care Jena (SBBS), Jena, Germany; 4 University Hospital Jena, Institute of Physiotherapy, Jena, Germany

**Keywords:** Report on interprofessional learning, Interprofessional learning in vocational education, Interprofessional collaboration in health professions, Interprofessional collaboration between medical students and students in nursing and physiotherapy, Video-based self-reflection, Video-based self-assessment, Role play, ideo-based role play

## Abstract

**Introduction: **Interprofessional collaboration is crucial to the optimization of patient care.

**Aim: **This paper aims to provide recommendations for implementing an innovative constructivist educational concept with the core element of video-based self-assessment.

**Methodology: **A course for students in medicine, physiotherapy, and nursing was developed through interprofessional, cross-institutional collaboration. The course consisted of

1. drawing on prior knowledge about the work done by each professional group in regard to a specific clinical scenario and an interprofessional treatment situation,
2. filming a role play of this treatment situation, and
3. a structured self-assessment of the role play.

We evaluated the preparation and implementation of the three courses conducted thus far. Concrete recommendations for implementation were made based on evaluation sheets (students), open discussions (tutors, instructors, institutions) and recorded meeting minutes (project managers, project participants).

**Results:** Basic recommendations for implementation include: selecting appropriate criteria for self-assessment and a simulated situation that offers members of each professional group an equal opportunity to act in the role play. In terms of administrative implementation we recommend early coordination among the professions and educational institutions regarding the target groups, scheduling and attendance policy to ensure participant recruitment across all professions. Procedural planning should include developing teaching materials, such as the case vignette and treatment scenario, and providing technical equipment that can be operated intuitively in order to ensure efficient recording.

**Conclusion: **These recommendations serve as an aid for implementing an innovative constructivist educational concept with video-based self-assessment at its core.

## Zusammenfassung

**Einleitung:** Interprofessionelle Zusammenarbeit ist eine unabdingbare Voraussetzung für die Optimierung der Patientenbehandlung.

**Zielsetzung: **Ziel dieses Erfahrungsberichtes ist es, Empfehlungen zur Implementierung eines innovativen konstruktivistisch geprägten Lehrkonzepts mit dem Kernelement der videobasierten Selbstreflexion zu geben.

**Methodik:** Im interprofessionellen, interinstitutionellen Diskurs wurde eine Lehrveranstaltung (LV) für Auszubildende der Gesundheits- und Krankenpflege und der Physiotherapie sowie für Medizinstudierende entwickelt. Die LV beinhaltete

1. die Wissensreaktivierung zum Tätigkeitsprofil der beteiligten Professionen, dem Krankheitsbild und einer interprofessionellen Behandlungssituation,
2. die Durchführung eines videodokumentierten Rollenspiels zu dieser Behandlungssituation und
3. die strukturierte Selbstreflexion des Rollenspiels.

Die Vorbereitung und Durchführung der drei bisher durchgeführten LV wurde evaluiert. Anhand der Evaluationsbögen (Auszubildende, Studierende), offenen Diskussionen (Tutoren, Dozierende, Institutionen) und protokollierten Treffen (Projektverantwortliche, Projektbeteiligte) wurden konkrete Empfehlungen zur Umsetzung abgeleitet.

**Ergebnis: **Wesentliche Empfehlungen

1. zur didaktischen Umsetzung sind: Auswahl einer geeignetem Behandlungssituation mit ausgewogenem Handlungsanteil der beteiligten Berufsgruppen für das videodokumentierte Rollenspiel und Erarbeitung von Kriterien zur Strukturierung der Selbstreflexion;
2. zur administrativen Umsetzung: frühzeitige interprofessionelle/interinstitutionelle Abstimmung bei der Festlegung der Zielgruppen, der Zeitplanung und der Verbindlichkeit der LV zur Sicherstellung der ausgewogenen Teilnehmerrekrutierung;
3. zur Ablaufplanung: Erstellung von Unterrichtsmaterialien, wie Fallvignette und Behandlungsszenario und Bereitstellung intuitiv bedienbarer Präsentationsmedien, um einen effizienten Videodreh zu ermöglichen.

**Schlussfolgerung: **Aus unserer Sicht stellen diese Empfehlungen eine konkrete Hilfestellung zur Implementierung des innovativen konstruktivistische geprägten Lehrkozepts mit dem Kernelement der videobasierten Selbstreflexion dar.

## 1. Introduction

**Progress in the medical field has been contributing to a rapid increase in the complexity and specialization of patient care** [1]. Specialization leads to a higher quality of treatment across all dimensions of patient care: treatment by physicians and nurses, as well as simultaneous care from other professionals, such as physiotherapists [2]. However, specialization also leads to more fragmented patient care [3]. Currently health care providers from different professions mostly act independently [4], [5]. Acting independently increases the risk of problems at the interface between the different health care professions and the health care providers such as physicians, nurses and physiotherapists. Such problems may have an impact on the over-all quality of health care. At best the positive effects of specialization are not fully exploited; at worst, it can lead to severe complications for the patient [6].

**Reducing interface problems through interprofessional collaboration requires awareness of the problem** [7], [8]. From our perspective, awareness should be raised from the very beginning of professional education. During this phase of training, interprofessional collaboration must be learned and practiced. At the same time, a professional attitude towards other professional groups must be developed. Attitudes are usually the result of personal experience. Therefore, changes in attitude are made on the basis of personal experiences, but are rarely the result of a cognitive process. Changes in personal and professional attitudes can be achieved through effective constructivist learning [9]. In this context, instructors are not necessarily charged with the task of transferring knowledge, but rather act as facilitators by creating an open learning situation, assigning defined tasks and providing materials, techniques, and experiential knowledge [10].

The main areas in interprofessional teaching and learning are

1. knowledge of the responsibilities (potentials and limitations) of one’s own and the cooperating professions,
2. mastery of occupation-specific and cross-occupational competencies and
3. adopting and exhibiting a professional attitude as the foundation for respectful interaction on equal footing [11], [https://www.cornelsen.de/bgd/97/83/06/45/03/20/5/9783064503205_x1SE_S138-143.pdf].

Ideally, the necessary knowledge, abilities, and attitudes for successful interprofessional collaboration are fostered during vocational education and university study through joint interprofessional courses [12]. However, this has yet to be set down in contemporary educational guidelines [13].

**An innovative teaching strategy for interprofessional education was developed at the Jena University Hospital in cooperation with the Vocational School for Health and Social Care (SBBS), also in Jena, Germany. Our educational concept is based on a constructivist approach **[14]. This approach includes drawing on and communicating prior knowledge to create a common knowledge base for all participants, experiencing a typical learning situation with key features, and reflecting on what has been learned [15], in our case through video-recorded role playing [16], [17]. Following this approach, we designed a course on interprofessional collaboration.

### 1.1. Aim

The goal of this paper is to provide recommendations based on our experiences for implementing this approach to interprofessional education using video-based self-assessment.

## 2. Issue

When developing this educational concept into a course, we raised curricular, administrative and process-based questions both prior to and during implementation. In this paper, we wish to share key insights on successful implementation of this educational concept.

## 3. Methodology

### 3.1. Description of the course

The constructivist approach [18], [19] includes drawing on prior knowledge, learning and reflecting [20]. Based on this, we developed a course to promote the acquisition and development of interprofessional skills. A core element of our teaching strategy is criteria-based, multi-dimensional self-assessment with video-recorded role playing in a safe learning environment. Through role playing and the video-based self-assessment, participants had the opportunity to confront their own attitudes and (inter)professional actions while collaborating with members of other health professions.

Turning the concept into a course required interprofessional and cross-institutional coordination and the development of four areas: curriculum, administration, procedural planning, and evaluation.

***Curricular coordination*** included the definition of learning objectives and content, as well as teaching methods.

**Learning objectives: **We defined** three general* learning objectives*** for participants:

1. describing the tasks of one’s own and other professions and explaining the occupation-specific relevant aspects of the clinical picture(knowledge),
2. acting and communicating in an interprofessional manner appropriate to the situation (application), and
3. reflecting on one’s attitude and actions in interprofessional situations (reflection).

**Instructional content: **A ***practice-relevant situation*** was selected for the recorded role play; in this case, the situation simulated the initial mobilization of a stroke patient and subsequent handover. For this purpose, a case vignette (see table 1 (Tab. 1)) was developed with regard to all subject areas. The necessary ***prior theoretical knowledge*** was defined to conduct the role playing activity: knowledge of the basic tasks of each profession, knowledge of the clinical picture and the necessary diagnostic and therapeutic measures from the perspective of each profession. In addition, criteria were set down in advance for ***self-assessment*** and structured analysis of the video-recorded role play.

**Teaching methods: **To call forth prior knowledge, the treatment situation, the video-based self-assessment and procedural plan (see Attachment 1) were designed and implemented as follows: 

In order to **reactivate prior knowledge,** we designed a small-group activity. To gather the occupation-specific prior knowledge, participants of the same professional group were asked to list the tasks and functions of another professional group and to then present these in a large group discussion. The participants from the other professional groups were able to add information as needed. To call forth occupation-specific prior medical knowledge, a plan identifying the problem, objectives and actions was developed in monoprofessional small groups and then presented in interprofessional group discussion.

To **design the simulated scenario**, we used a case vignette to define the occupation-specific tasks within the*** learning situation***, the amount of detail for the “stage directions” for role playing, and the framework of implementation (participants assuming not only their own professional roles but also a different one, discussing the results of the simulated scenario in the context of a handover).

For the **multi-dimensional self-assessment**, we chose the format of a moderated group discussion. The discussion was preceded by a phase of individual work in which students were able to map out their perceptions of the learning situation using a multi-dimensional list of criteria for action, interaction, and communication.

**Cross-institutional administrative coordination** included the coordination of all necessary measures between the participating institutions. This concerned the selection of the professional groups, the desired level of experience (medical students in their sixth year of study and advanced vocational students), the attendance policy for the course (required), the appropriate timeframe for course [16 course units on two consecutive days], as well as scheduling.

**The coordination of procedural planning **included planning the details for practical implementation (blueprint: lesson planning, teaching materials, participant grouping, instructor competencies, supervision, participant numbers and group distribution, technical equipment and medical props, see Attachment 2).

The **evaluation** included selecting evaluation instruments (UWE-IP questionnaire and the faculty evaluation sheet) and developing an individual questionnaire to better capture participants’ subjective experiences).

#### 3.2. Generating recommendations

The recommendations described here result from the intense preparation and continual optimization of three cycles of the course with a total of 54 participants. The first course included 28 units and two simulated scenarios (initial mobilization post-stroke and post total hip endoprosthesis [TEP]) for the video-recorded role playing. The course was continually improved through an iterative process. The course was shortened to 16 units and focus was placed on a single complex simulated situation, instead of on two. The optimization process followed the PDCA (plan-do-check-act) principle, the **key concept of quality management**. In the meetings held before and after, requirements were compiled for each aspect relevant to implementation. Afterwards, the plan was drafted (“plan”), measures were implemented (“do”), evaluated after each round and compared in terms of target/performance analysis (“check”), and the necessary improvements were identified (“act”).

Each of the three courses was evaluated extensively by both participants and instructors. The participants evaluated the course using pre-defined or specially developed evaluation instruments, while the instructors evaluated the sessions in semi-structured, open discussions. We derived recommendations for curricular and administrative implementation, procedural planning and evaluation based on the evaluation results (students), the open discussions (tutors, instructors, educational institutions), and the recorded minutes of meetings (project managers, project participants).

## 4. Results: recommendations for implementation

### 4.1. Recommendations for curricular implementation

**Defining learning objectives and contents and selecting teaching methods occurred as part of curricular planning.**

#### Learning objectives

***Requirement: ***The interprofessional learning objectives must be clearly defined and communicated to the participants in advance.

***Experience and recommendation***: Based on the general learning objectives, we defined specific learning objectives in the following areas: occupational knowledge and basic communication, pathophysiology, diagnostics, and therapeutic measures for the selected clinical case in a professional and interprofessional context. The definition of the learning objectives was taxonomically refined and enhanced throughout the course. Further specification of the learning objectives enabled both participants and instructors to experience the video-based self-assessment as being even more constructive.

We recommend defining the following specific learning objectives. 

Participants should be able to

1. describe the range of competencies of their own and the other professions,
2. understand the clinical case and treatment scenario and explain them competently and accurately from the perspective of their own profession,
3. develop their own professional problem-objectives-measures plan,
4. act and communicate interprofessionally and in a manner appropriate to the situation,
5. recognize, name, and communicate errors in the intra/interprofessional treatment process,
6. conduct a patient-centered, disease-specific handover between professional groups, and
7. reflect on their own attitude and actions.

##### Instructional content and learning situation

***Requirement: ***The educational situation and the resulting occupation-specific learning content should be selected with the specific interprofessional learning objectives, participants and their prior experiences in mind. The learning situation should reflect an authentic, complex scenario relevant to practice that facilitates interaction between the professional groups and with the patient.

***Experience and recommendation:*** In the first three sessions, two treatment situations were used for the role-play activity: early mobilization of a patient who was subjected to a hip-TEP and the initial mobilization and rehabilitation of a patient in a post-stroke state. These situations were chosen because the necessary interprofessional collaboration for the initial mobilization of a patient requires coordination and close cooperation among the care givers. During role playing and the subsequent assessment, many participants experienced moments of revelation (see Attachment 3) as they viewed themselves on the video. Participant and instructor evaluations, however, revealed that the hip-TEP role-play activity was seen as less suitable due to the greater importance of physiotherapeutic measures as opposed to other forms of care. For this reason, we recommend ensuring that all professions are equally involved in the simulated scenario.

##### Teaching methods

***Requirements: ***Teaching methods should be selected based on the learning objectives, content, the knowledge base and prior experiences of the participants. Activating existing knowledge, expanding on that knowledge and facilitating the reflection process, each o these goals requires the application of different methods. It is important to select teaching methods that are student-centered and not instructor-centered.

***Experience and Recommendation:***

**Activating prior knowledge: **We developed a small-group activity designed to activate prior cognitive knowledge. Instead of listening to a lecture, participants in monoprofessional groups identified the problem and, drawing on their theoretical knowledge and previous practical experiences, developed a plan that reflected the objectives and actions for the selected clinical case (stroke). Both the occupation-specific and the interprofessional measures were discussed and expanded on in a large group discussion and then combined into an overall plan for taking action. Initially, we had selected a lecture block format with six units to teach the clinical, physiotherapeutic and nursing knowledge relevant for both clinical cases. However, discussions with the participants revealed that the lecture format was perceived as tedious, rather than helpful. In contrast, the development of a problem-objectives-measures plan enabled students to apply their prior knowledge to the specific clinical case. This stimulated the interprofessional discussion among the participants. For this reason, we highly recommend a student-centered format to tap into prior knowledge.

To re-activate specific skills and impart the ability to engage in interprofessional interaction, we encouraged peer-teaching among the participants during the preparation for the video-recorded role play. This instructional style was very well received by the participants and is therefore recommended 

**Learning experience:** A core element of a constructivist instructional approach is facilitating learning in a specific situation: in our case, a documented small-group simulation of a hospital scenario, initially without and then with an exchange of roles. We also conducted a role-play activity simulating an interprofessional patient handover during a large group discussion. Participants were divided into small interprofessional groups of four to five participants, with at least one student from each professional group. The participants received the case vignette, the treatment scenario, and the assignment to create a video demonstrating the ideal procedure (e.g. an “instructional video”) for interprofessional collaboration in this situation. To make the treatment situation more realistic, it was based on the previously developed problem-objectives-measures plan. Participants worked independently on how to present the treatment situation. For instance, they could choose whether they wanted to practice individual steps of giving treatment before taping, or record the entire situation without rehearsal. In the first round of the role play, participants assumed their own professional roles as physician, physiotherapist or nurse, with another participant acting as the patient, and yet another participant recorded the video. In the second round and the second session (after improvements were made), the participants exchanged professional roles giving all a chance to experience the scenario from a different vantage point. We recommend holding the role play twice to allow the participants to become acquainted with the situation from the perspective of their own profession and that of another.

**Reflection: **One crucial part of this teaching strategy is to foster extensive reflection by the participants on their performance. The task of the teacher is to discreetly facilitate the discussion from the background, without disrupting the participants’ articulation of their experiences.

To achieve **structured reflection**, we developed a list of criteria based on our experiences (see table 2 (Tab. 2)). We recommend considering three categories: action, interaction, communication. Each participant was asked to reflect on whether the quality of the treatment given met the standards of care (action), whether the collaboration with their interprofessional colleagues was effective (interaction), and whether their communication with patients and interprofessional colleagues was respectful and on equal footing (communication). In the larger group, participants were given the opportunity to freely express their feelings about their personal experiences and perceptions. Everyone involved in the simulation, as well as the observers, were invited to participate in the discussion to identify potential differences in perception of the situation.

In contrast to the open discussion style used in the first round, it turned out that criteria-based and structured reflection according to categories has clear advantages, although it should be limited to key aspects. It is impossible to address all aspects of a complex action from all perspectives and across all categories. Therefore, we recommend that the discussion moderator facilitate and encourage an in-depth discussion, while at the same time structuring the discussion to ensure that the allotted time is not exceeded.

#### 4.2. Recommendations for administrative implementation

**The basis for successful administration is careful cross-institutional coordination of all basic curricular, administrative and procedural aspects.**

##### Cross-institutional coordination

***Requirement: ***All administrative and curricular aspects should be agreed on and jointly planned with all institutions early on and step by step in more detail over time. It is imperative that these aspects can be integrated into the curriculum and schedule of each educational program.

**Figure 1:**
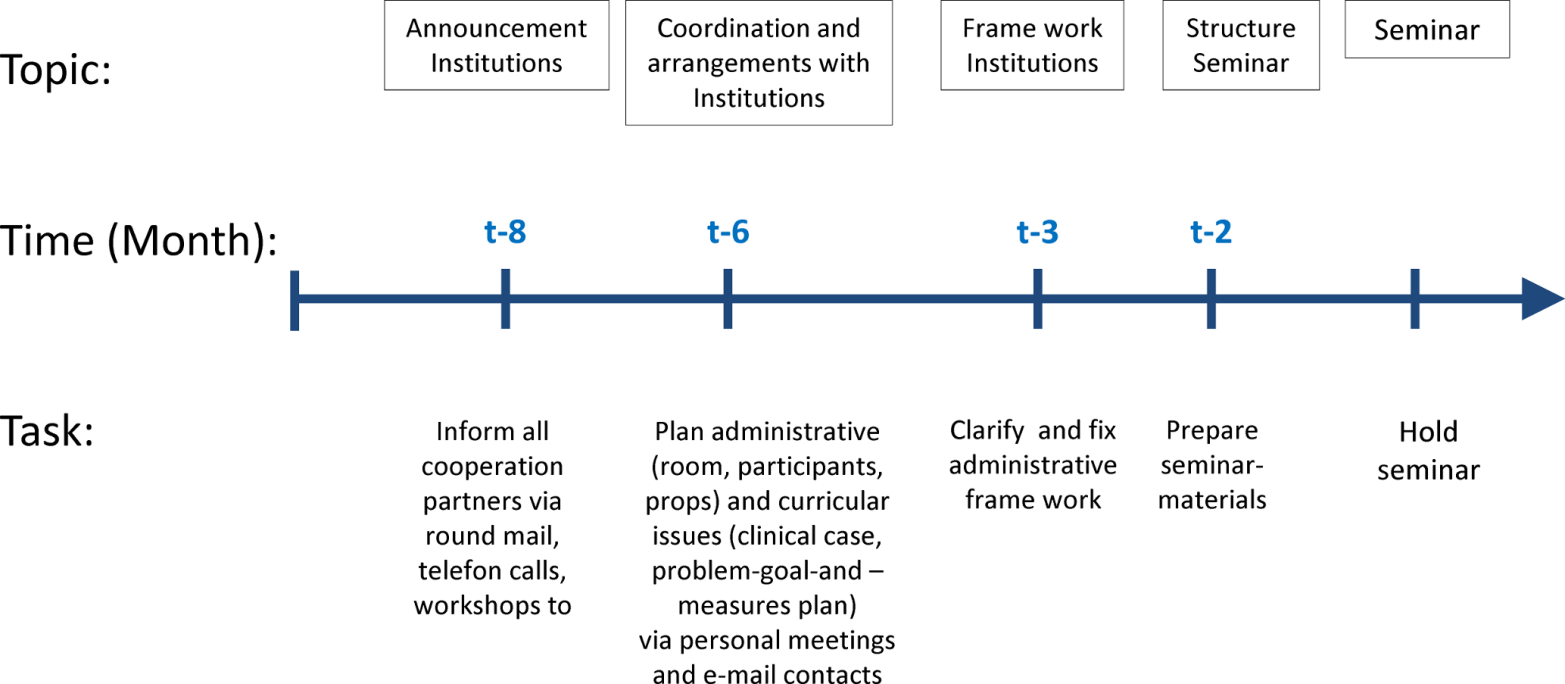
Time planning. Interinstitutional Coordination and Process planning.

***Experiences and recommendations: ***Three educational institutions were involved in our project: the senior nursing staff management at the Jena University Hospital, the academic administrators of the SBBS, and the Dean of Studies at the Medical School. To develop the basic outline of the course, we held discussions with the staff and decision-makers at the institutions involved, as well as with the department heads in the relevant medical disciplines (neurology, geriatrics, physiotherapy). Main topics included the selection of the professional groups and the length of the course. The length of the course dictates the requisite leave of absence from practical duties and the extent to which other courses need to be changed. Just as important was the coordination of schedules in order to avoid conflicts with tests, exams or other mandatory courses. We also met with staff from the institutions involved to coordinate other important details, such as room scheduling, technical equipment and medical props. This process began five months before the first course session and continued over multiple team meetings. The planning of the subsequent courses was much simpler: this merely required telephone calls with the staff in charge and one or two team meetings (see figure 1 (Fig. 1)).

Based on these experiences, we recommend starting the cross-institutional coordination and procedural planning at least six months prior to the first course session.

Considerations regarding the selection of professional groups (occupation and educational level), the attendance policy for the course, the selection of an appropriate timeframe, and scheduling are closely interconnected. In order to provide a clear overview, we present these considerations separately.

##### Target group selection and recruitment

***Requirement: ***The target groups should be determined during the first planning stage. Based on the definition of interprofessionalism at least two professions should be involved, although three professions are preferable: physicians and members of the health professions. Identification of the requisite educational level should take place at the beginning of the planning phase. Ideally, the course should target participants with some experience in interprofessional contexts.

***Experiences and recommendations:*** The decision regarding the three target groups of nursing professionals, physiotherapists, and physicians was done prior to entering the planning and preparation stage. This decision determined the selection of the two clinical cases and other learning content. Making these decisions early proved to be crucial and is highly recommended because they serve as the foundation for all subsequent planning and preparation. The involvement of three professional groups has also proven to be valuable and is recommended.

Based on the types of professional groups and the focus on mobilizing a patient in the context of early post-stroke rehab, we sought to involve participants with basic experience in patient care and treatment. In cooperation with the nursing staff management at Jena University Hospital and the administrators at SBBS, we decided to involve second-year students from the health care and nursing program and from physiotherapy. Starting with the second cohort, the course was offered as a mandatory course for medical students in their sixth of formal study. We recommend recruiting participants at a more advanced level, since sufficient prior experience is needed for meaningful participation in discussions.

##### Attendance policy and scheduling

***Requirement: ***The duration of the course and whether it is optional, elective or mandatory should be determined in the early planning phase in compliance with program rules and regulations.

***Experience and recommendations: ***At first, the course was offered as an elective for medical students in their eighth and tenth semester with 28 units on five different days. It became evident that unavoidable scheduling conflicts on several days of the course made attendance by the medical students impossible due to the very individual nature of students’ course schedules. In contrast, the course was part of the mandatory coursework for students in the nursing and physiotherapy programs since these programs do not have elective courses.

As a result, the course was shortened to two days and offered as a mandatory course for sixthyear medical students in geriatrics, neurology, and physiotherapy. This decision turned out to be very reasonable not only in respect to ensuring attendance, but also to the desired level of professional experience. Therefore, we recommend designing the interprofessional course to be as compact as possible and integrating it into the required curriculum to lower the incidence of scheduling conflicts and reduce the need for extra organizational efforts.

##### Scheduling the course as part of the overall curriculum

***Requirement: ***Scheduling the class must be done in close coordination with all institutions, study programs, and instructors and should take place in the very early phase of planning.

***Experience and recommendation:*** Immediately after scheduling and recruiting the instructors, we started determining the target group and the duration of the course. The first offering of the course was held as part of the theoretical phase of the vocational programs, which turned out to be suboptimal for the health care students since they had to spend time catching up on the regular course content. After a discussion with the instructors, subsequent courses were held during the practical phase of study. Since scheduling had to accommodate the practical phases of two vocational programs, a planning time of four to six months proved valuable. The instructors also considered found this time period appropriate. For this reason, we recommend implementation during the practical phase of the vocational programs with the requisite long-term planning.

#### 4.3. Recommendations for procedural planning

**Procedural planning requires the determination of all details of practical implementation.**

##### Planning course content

***Requirements: ***Planning and developing content for the course sessions, as well as concrete lesson plans should be done after initial cross-institutional coordination and take place in interprofessional dialogue. Course planning should be continuously optimized.

***Experiences and recommendations: ***We determined the learning objectives and teaching tools for the individual sessions during interprofessional project meetings. Concrete details were planned by the staff involved with the project. The resulting plans were discussed in the project meetings and modified if necessary.

To optimize the course based on the PDCA cycle, all content-related and procedural details, as well as participant feedback (participants, instructors, tutors) were examined critically and used as the basis for improvement. The results were incorporated into the planning of the subsequent semesters.

This kind of critical interactive work made it possible to quickly identify and address weak points, such as time management for certain units (e.g. reduction of the time provided for group discussions about occupations), the comparison of instructional content with prior knowledge and experiences of the participants (e.g. reduction of content on basic communication), the extent of tutor intervention during role playing (e.g. reduction of support and correction through tutors).

We recommend reserving time for multiple sessions to prepare the initial course. For later optimization, we recommend two meetings: one to analyze the previous session and determine changes and another to approve planned changes.

##### Participant numbers and distribution 

**Requirement: **The desired number of participants should be determined before the course is offered since it determines the required resources (instructors, teaching assistants, rooms, props) and the number of groups for the group activities. When forming small groups, it should be ensured that each profession is represented.

***Experiences and recommendation: ***We limited the number of participants to a maximum of 15 per course (five students from each profession) to create the space for cooperative collaboration and in-depth group discussion. The videos were recorded in small groups of four to five participants (“physician”, “nurse”, “physiotherapist”, “patient” and “director/cameraman”). Participants responded positively to the numbers and groupings. For this reason, we recommend this size and composition for the small groups.

##### Teaching materials

***Requirement: ***The instructional materials aimed to prepare participants for the simulated scenario and to support them in the role play and their reflections on it.

***Experience and recommendations: ***We designed the following instructional materials: case vignette, treatment scenario and assignments for compiling the tasks and responsibilities of specific collaborating professional groups, experiences in interprofessional collaboration, problem-objectives-measures plan, recording, and criteria for reflection. We will now describe the materials and the sequence of their use in the teaching sessions.

To address the topic, we invited participants to report on their own interprofessional experiences and to share their ideas about the other two professional groups in the form of written assignments.

To prepare participants for the treatment scenario, we developed a case vignette (stroke patient) in collaboration with a neurologist. In monoprofessional small groups, the participants received the task of creating a problem-objectives-measures plan for the clinical case of a stroke patient. Based on this task, they were asked to review their knowledge and apply it to the case. The results of all three small groups were shared in a large group discussion to establish an overview of the situation. Their performance showed that the participants collectively possessed solid prior knowledge that they were able to apply. This assignment was evaluated positively by the participants.

Prior to recording their video, participants received a short description of the treatment scenario with an overview of the individual steps to be taken and the assignment to create an ideal “instructional video” within 90 minutes. The resulting videos were sound in terms of quality and demonstrated a great degree of interprofessionalism in the interaction between the professional groups. During this intense and highly motivated small-group activity, spontaneous “cross-peer-teaching” took place for the purpose of giving instructions. We were able to observe in-depth and supportive collaboration in interprofessional dialogue.

##### Instructors and tutors: number, competency and tasks

***Requirement: ***Instructors should be selected with respect to their expert knowledge and competency, as well as their communication and media skills. They should be assisted by well-trained tutors. The number of instructors and tutors should be adjusted for the small-group format.

***Experience and recommendation: ***We developed the course as a team consisting of a representative from physiotherapy, academic administrators at SBBS, an expert in pedagogy, a physiotherapist, and nursing professionals in cooperation with additional experts in neurology, orthopedics and psychology. The implementation of the course required one instructor for each session to lead the large group activities and discussions. The instructor was supported by a tutor who, for instance, wrote down comments made during discussions, operated technical equipment for presentations, and supported small-group activities, especially filming the role-play activity in the case of medical or technical questions).

To implement a course of this nature, we recommend involving at least one instructor and one tutor. Determining the appropriate amount of intervention during the role play was of the utmost importance. A complete lack of intervention in the case of incorrect actions during interprofessional teamwork leads to incorrect engrams. Too much intervention disrupts the participants’ creative process during their engagement with the content and interprofessional dialogue. For this reason, we recommend that instructors and tutors assume the following tasks: moderating group discussions based on their own knowledge and communicative abilities, supporting small-group activities upon request and in the case of technical difficulties.

##### Classroom space

***Requirement: ***The specific requirements of the instructional methods must be taken into account when planning the required space.

***Experience and recommendations: ***Spatial requirements depend on the teaching method (short presentation or group activity with role playing for the practice-relevant treatment situation, filming and the number of participants). Since we conducted role-play scenarios in three small groups, we needed three clinical nursing labs with basic equipment (hospital bed and small medical props). A seminar room with technical equipment was needed for the short presentations and group discussions. The initial conferences about classroom requirements (seminar room, nursing equipment) and their availability occurred among the participating educational institutions about six months before the course was held. Against this backdrop of cross-institutional coordination, the facilities were examined in terms of their suitability (number of adjacent rooms, size, equipment, lighting, noise level).

Due to the high demand for classroom space and the resulting difficulty in obtaining these spaces, we recommend discussing the issue of space as early as possible, via resources such as SkillsLab, practice facilities at medical schools or nursing labs at vocational schools.

##### Media technology, presentations and filming

***Requirements: ***Electronic devices for recording videos and giving presentations should be easy to operate so that technical difficulties are ruled out. Each instructor and tutor should be able to operate the devices without problem.

***Experiences and recommendations: ***We used a laptop and projector for lectures and short presentations, as well as tablet for filming videos. For the self-assessment and reflection on the videos in the large group discussion, we connected the tablets to the laptop and presented them using the projector amplifying the sound with additional loud speakers. The use of tablets enabled us to play the videos on a large screen and watch particular scenes multiple times in order to best evaluate them. This was highly appreciated by the participants. At the beginning we used digital cameras with a small screen which were much more difficult to operate. In addition, it was more difficult to show the material immediately.

For this reason we recommend the use of tablets to film role-play activities.

##### Props

***Requirements:*** Authentic representation of practice-relevant treatment situations should be made possible through the use of real, hospital-sourced equipment. These materials should be acquired in a timely manner, and their acquisition should be discussed with the people in charge.

***Experience and recommendation:*** In addition to the hospital bed including sheets, we used occupation-specific materials, such as a stethoscope, blood pressure cuff and a reflex hammer. We also used professional and patient clothing to distinguish between the professions and to identify the patient. We recommend the use of original aids and equipment from clinical practice for authentic representation of practice-relevant treatment situations.

#### 4.4. Evaluation

***Requirements: ***Educational interventions should be s to be evaluated [21]. The evaluation was designed to assess the curriculum, administrative organization, procedural planning, and the personal benefit for each participant. The evaluative efforts should be in keeping with the significance of the course and mindful of existing resources.

***Experiences and Recommendation: ***Each repetition of the course was evaluated extensively by both the participants and instructors. We obtained helpful information by administering our own semi-structured questionnaire to participants. In this questionnaire, we provided space for open-ended responses about certain aspects of the educational concept (see Attachment 4). This allowed us to ask for specific information about the experience of role playing, such as any moments of revelation, and how the participants experienced their own actions from the perspective of another professional group. During the first offering of the course, we only used previously existing questionnaires for the purpose of participant evaluation (University of Western England Interprofessional Questionnaire (UWE IP) [http://www.klinikum.uni-heidelberg.de/UWE-IP.136337.0.html], the University of Jena’s Instructor Evaluation Sheet). However, neither of these questionnaires covered the experience gained during the course or the experience of the learning situation.

The evaluation by the instructors was conducted in a structured and recorded discussion after each daily session and after completion of the course. The approved suggestions were implemented in the future planning of course sessions to the extent possible. Changes included shortening of the course and changing recruitment requirements and making alterations to lesson plans, as previously discussed.

We highly recommend a critical 360° evaluation and interactive procedures based on the PDCA cycle. For this reason, we recommend the development and use of individual semi-structured questionnaires to solicit responses from the participants. We recommend structured open discussions for team evaluation. By doing this, critical issues that require action can be quickly recognized and dealt with.

## 5. Discussion

We have derived recommendations from our experience with this course that can be applied to other interprofessional teaching situations following a constructivist approach. Our approach enabled participants to gather experiences about their own professional skills through role playing and reflecting on them based on the videos made of the role plays. By doing this, we sought to provide participants with a positive interprofessional experience and support them as they incorporated this experience into their professional routine.

**The constructivist approach has been valuable in teaching interprofessional skills.** Perceiving one’s attitude in an interprofessional situation and changing one’s attitude through reflection on one’s actions are more complex processes than the cognitive acquisition of content. Fulfilling this learning objective can be best achieved by a constructivist approach to teaching and learning, one that is frequently used in interprofessional teaching [10], [14].

**Learning by doing requires an appropriate and controlled learning environment.** Based on our experience, the selection of an appropriate learning situation is the decisive factor for a sustainable learning experience. This insight is also confirmed by other working groups [22], [23]. Participants respond to a well-chosen situation with a willingness to engage in interprofessional learning and a willingness to change their professional behaviors [22], [23]. Participants were given the opportunity to experience themselves and their own actions through the eyes of their own profession and those of another profession.

Our participants reported an increase in the moments of revelation when they changed perspectives. In the scientific literature, changing perspectives during role play is hardly discussed [24], [25]. The results of our evaluation clearly show that participants found this element to be particularly instructive. The perception of their own, sometimes helpless actions from the perspective of a different professional group increased their understanding of the complex demands placed on other professions and an understanding of the associated challenges. This experience was seen as helpful for developing a respectful attitude.

**Learning is supported by conscious reflection on one’s own actions. **We sought to intensify the self-reflection by documenting the role-play activity on film.

We used video recording as a means for participants to experience and reflect on their actions in the role of their own and another profession. Using video recordings facilitated the criteria-based, structured reflection since key scenes could be reviewed repeatedly for more precise analysis, if needed. The participants could watch themselves and witness their effect on the others (self-perception), and they received feedback from their colleagues (perception by others).

Video recording has not been used extensively in medical education. Its use varies greatly: Instructional videos are often shown to teach practical skills and demonstrate ideal procedures [26]. In interprofessional education, videos with intentional mistakes have been used to stimulate discussion. Hyer et al. used video recordings of simulated interprofessional team meetings to teach participants how to recognize effective interprofessional interactions [20], [26].

However, video recordings are not typically used to recognize one’s own mistakes and to develop strategies to avoid them. Based on our positive experiences with the routine use of video recordings of each activity during the teaching of surgical skills [27], we adapted this procedure to the interprofessional teaching context.

Positive experiences and student feedback have encouraged us to recommend and use this teaching tool more widely in interprofessional teaching and learning.

## 6. Conclusion

In our hands the constructivist approach was very successful in supporting and reinforcing the development of interprofessional skills. Drawing on our experience, we have made recommendations for transferring this strategy to other interprofessional teaching situations. In our opinion, these recommendations are also helpful for implementing video-based self-assessment as a teaching tool in interprofessional courses.

## Funding

The project is received a grant from the Robert Bosch Stiftung (project number 32.5.1316.0008.0).

## Competing interests

The authors declare that they have no competing interests.

## Supplementary Material

Detailed course plan for a selected workshop day (day 1)

Blueprint of the workshop (in blue our implementation)

Excerpt from the evaluation results

Evaluation tool

## Figures and Tables

**Table 1:**
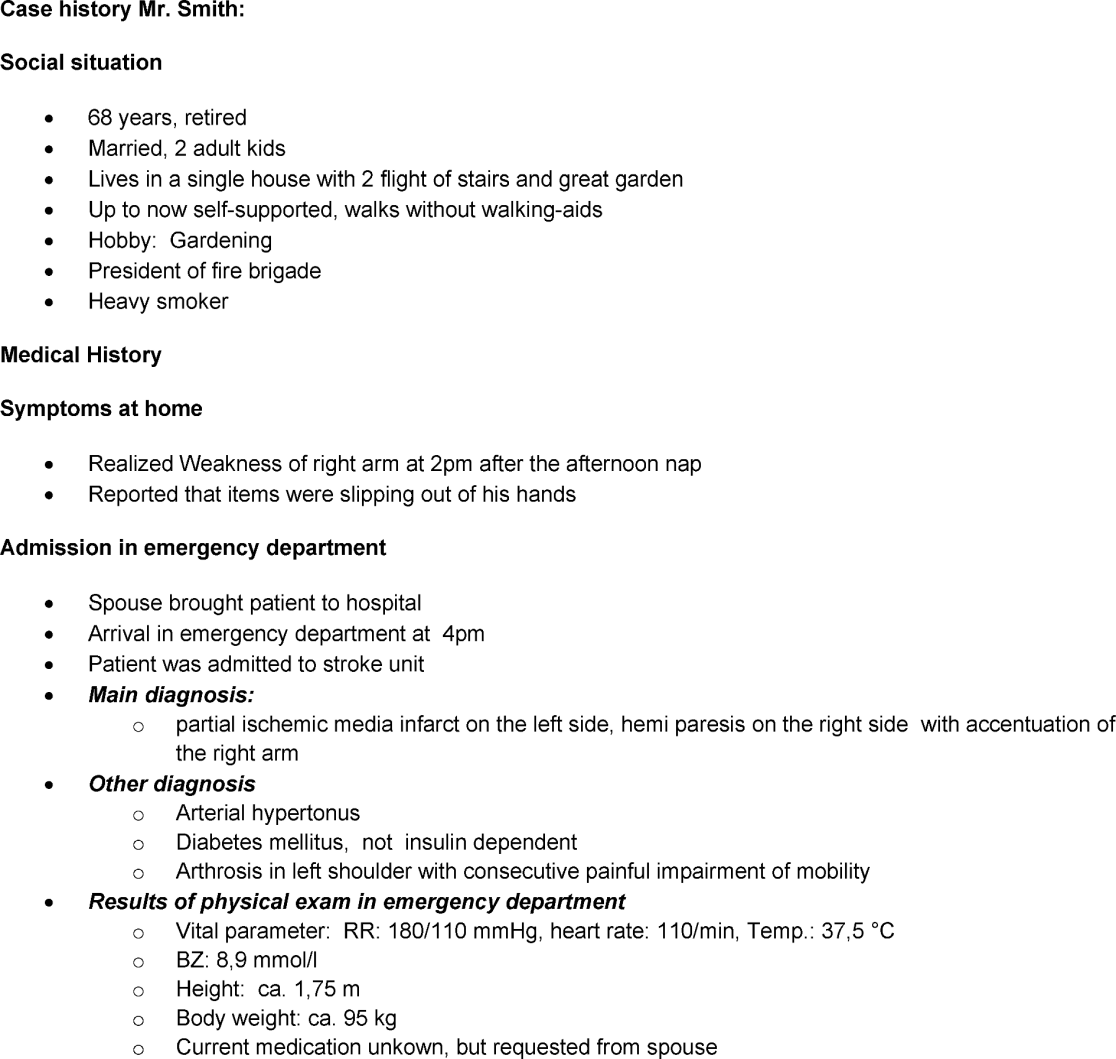
Case vignette Stroke

**Table 2:**
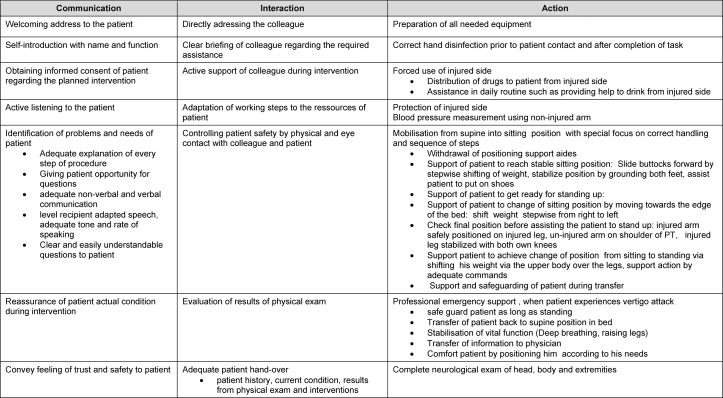
Analysis criteria for role play according to key steps
