# Supplementary material for: Recommendations to enhance constructivist-based learning in Interprofessional Education using video-based self-assessment
Source: GMS J Med Educ. 2016 Apr 29;33(2):Doc33. doi: 10.3205/zma001032 (PMC4895861; doi:10.3205/zma001032)
Supplement: Detailed course plan for a selected workshop day (day 1) [file JME-33-33-s-001.pdf]

	Content	Method / Sequence of steps / *Media*	Time frame	
**Introduction**<br><br>**PROFESSIONs:**<br><br>**BLAU: PHYSICIAN**<br>**ORANGE: NURSE**<br>**GRÜN: PHYSIO**<br><br>Instructors:<br>1, 2, 3, 4, 5	**Introduction of the workshop:**<br>• Introduction instructors<br>• Goals of interprofessional education<br>• Structure of workshop<br>• Educational goals (Bosch)<br>• Previous experience with videos in teaching?<br><br>**Introduction participants (P): n=13**<br>• Name, profession, eduational level<br>• Experiene in interprofessional cooperation<br><br>• Expectations for workshop	Presentation<br><br>Flipchart<br><br><u>Generate network: throw ball of wool and take picture</u><br>1st round: Give name, profession, educational level<br>2nd round: Give pos and neg experience with interprofessional cooperation<br>→ Gather resutls on flip chart<br><br>Distribute moderation cards (MC): B, O, G,<br>P record „central question“ and return MC to instructor, pin MC to flip chart and comment	**15**<br><br>**5**<br>→ 2-3 min. personal time<br>→gather results & comment<br><br>**20**	08:00–08:20<br><br>08:20–08:40
**Occupational Knowledge (Laws and regulations))**	• Name tasks<br>• Name responsibilities and fields of work according to profession	Moderation cards (B, O, G) / Flip chart:<br><br>Flipchart<br><br>**Task:** each profesion gathers for both respective other occupation the respective working fields → Instructors collects infomations on flip chart, Completion via profession in questions, discussion regardin potential overlap and differencs in working fiels and responsibility<br><br>Ex.: Physio, Physicians → present infos regarding responsibilities of nurses, Nurses add missing infos<br><br>**n=3 profession-specific small working groups**	**30**<br>→ 10 min. introduction<br>→ 5 min. Gather infor from MC on flip charts<br>→ 5 min. Discussion per Flipchart = 15 min	08:40-09:10

**COFFEE BREAK 15 min (10:00-10:15)**				
**PGM-Plan**	**Presentation of results from small working groups:**<br>• Profession specific<br>• Completion via other professions and instructor<br>• Structuring & summarizing of results	Moderation through instructor<br>Plenary discussion<br><br>Flipchart<br><br>**n = 3 small wrking groups**	**45**<br>15 min.<br>per group	10:15-11:00
**Video recording**<br><br>**profession-specific**<br><br>**Preparation**	**Introduction in video recording of role play:**<br>• Goals of this task<br><br>• Assigment of task: **Video of 2-4 min.**length<br>⇒ Role distribution: all 5 roles must be assumed, decision to be taken by P, => leading profession has to assume patient role<br>o **Nurse:** Preparation of patient. (Mobilisation from supine to sitting position, check of vital signs, drug application)<br>o **Physician:** neurological exam<br>o **Physio:** mobilisation from sitting to standing in front of bed<br>• Comments to using props<br>• Instruction to use tablet<br>• Assignment of rooms (Gruppen professionsbezogen)<br>• Preparation of video recording in small class rooms (Nursing lab): detailed Discussion of procedural plan	Introduction<br><br>Flipchart<br><br>**n = 2 small working groups, 2 videos**<br><br>Instruction of small working groups<br>P: Select props, distribute roles, prepare working area	**15**	11:00-11:30
**Video record**	Video record 2-4 min	Support as needed by instructors and tutors	**45**	11:30-12:15

**LUNCH BREAK 60 min (12:15 – 13:15 Uhr)**				
**Video analysis**	• Introduction and moderation<br>• Explain analysis criteria according to profession<br>360 degree reflexion (internal and external perspective) (other P and instructors) based on criteria list	Use criteria list<br>Demonstrate all 3 videos (Projector)<br>Plenary discussion	**60**<br>20 min.<br>per group +<br>video play &<br>view	13:15-14:15
**Video recording**<br><br>**interprofessionell with change of perspective**<br><br>**Preparation**	**Introduction in video recording:**<br>• Goals: change of perspective<br><br>• Assignment of task: **Video record 2-4 min.**<br>⇒ Role distribution: all roles must be assumed, decision taken by P<br>o **Nurse:** Preparation of patient, mobilisation from supine into sitting position, patient check<br>o **Physician: neurological exam**<br>o Physio: mobilization into upright position<br>• Comments to use of props<br><br>• Preparation of video record in nursing lab: Discussion of procedure in small groups	Introduction<br><br>Flipchart<br><br>Profession-specific group work:<br>→ Generate details of content: 3 small groups<br><br>Video record: distribution in small working groups<br>→ interprofessionell<br>→ Distribution per small working group	**30**<br><br>15 min.<br><br>15 min.	14:15-14:45
**Coffee break 14:45 – 15:00 Uhr**				
**Video analysis**	• Introduction and moderation<br>• Discuss criteria list<br>• 360 degree reflexion (internal and external perspective)	Analysis based on criteria list<br>Demonstration of all 3 videos (projector)<br>Plenary discussion	**60**<br>30 min.<br>per group<br>Video play &<br>view	15:00-16:00
**Colsing remark**	• Perspective<br>• Fare well		**2**	End around 16:05
