# Supplementary material for: Recommendations to enhance constructivist-based learning in Interprofessional Education using video-based self-assessment
Source: GMS J Med Educ. 2016 Apr 29;33(2):Doc33. doi: 10.3205/zma001032 (PMC4895861; doi:10.3205/zma001032)
Supplement: Blueprint of the workshop (in blue our implementation) [file JME-33-33-s-002.pdf]

Curriculum	Workshop
Instructional concept	Constructivism:<br>▪ Reactivation of previous knowledge, learning experience, reflexion
Educational goals	General goals<br>• describing the tasks of one's own and other professions (knowledge)<br>• explaining the occupation-specific relevant aspects of the clinical picture (knowledge),<br>• acting and communicating in an interprofessional manner appropriate to the situation (application),<br>• reflecting on one's attitude and actions in interprofessional situations (reflection)<br>• Identification of errors in intra/interprofessinal workflow and communication
Leanring situation	Selection of clinical case and treatment situation while ensuring an equal opportunity for participation of all professions<br>▪ Initial mobilisation of a stroke patient
Instructional method	*Reactivation of previous knowledge:*<br>▪ Presentation, small working group activities to gather occupational specific knowledge and to generate a problem-goals -measures plan
	Learning experience:<br>▪ Video documented role play of an interprofessional treatment situation (case vignette) in small working groups (initial mobilisation after stroke) with and without change of perspective<br>▪ Roe play of an interprofessional patient handover in the context of a case discussion in the large group
	Reflexion:<br>▪ Criteria-based<br>▪ Structured in communication, action and interaction
Evaluation	Questionnaire (participants) and structured discussion (project team and instructors)<br>▪ Standard evaluation questionnaire of the University hospital<br>▪ Specifically developed questionnaire<br>→ specific items, e.g. regarding the moments of revelation during the role play

Administration	Work shop
Identification of target groups	Definition of at least two target groups<br>▪ According to the announcement of the Bosch-Foundation: Mandatory to select physicians and two additional health care professions: nursing and physiotherapy
Interinstitutional coordination	Coordination between all involved institutions in respect to recruitment of participants, attendance policy, curriculum, scheduling<br>▪ Coordination between Nursing directory of the University Hospital, Head of the vocational School and the dean of the Medical Faculty
• Recruitment of participants	Definition of the requested level of experience<br>▪ Second year students from nursing and physiotherapy<br>▪ Sixth year medical students (interns)
• Attendance policy	Definiton of attendance policy (compulsory, elective)<br>▪ Mandatory class for nursing and physiotherapy students<br>▪ Mandatory class for medical students during internship
• Time Frame	Definition of the duration of the workshop in accordance with training regulation of the health care professions<br>▪ 2 days with 16 of training
• Scheduling of class	Definition of work shop times in accordance with the availability of instructors and participants<br>*Instructors:*<br>University Hospital Jena:<br>▪ Experimental Transplantation Surgery, Department of General, Visceral and Vascular Surgery,<br>▪ Institut for Physiotherapy<br>▪ Nursing Directory<br>▪ Dean of Medical Studies, Medical Faculty<br>▪ Department of Neurology<br>▪ Institute for Psychosocial Medicine and Psychotherapy<br>*Participants:*<br>▪ Nursing Directory, University Hospital Jena<br>▪ Directory of Vocational School for Health and Social Affairs (Berufsbildende Schule für Gesundheit und Soziales Jena (SBBS))

Procedural planning	Workshop
Procedural plan	Procedural plan for students and instructors<br>▪ Workshop times<br>▪ Sequnce of lessons<br>▪ Location<br>▪ Information material for all participants: General goal of work shop, date, location, duration, reading material, Special hints (what to bring such as professional clothing) ,<br>▪ Introduction of the work shop structure
Teaching material	Information material and working tasks<br>▪ Case vignette<br>▪ Problem-goals-measures plan<br>▪ Procedural plan: Over view regarding the modules and the clinical case<br>▪ Overview of procedural steps required for the patient and treatment scenario<br>▪ Criteria for video analysis (action, interaction, communication)
Participants:<br>• Number<br>• Group distribution	Teaching in small interprofessional working groups<br>*Number of participantsl:*<br>▪ 15 participants/Workshop with 5 students of each profession<br>▪ Group distribution for video recorded role play: 5 students per small group with at least 1 student from each profession<br>*Gruppenaufteilung:*<br>▪ In accordance with the role play distribution of participants in 2 – 3 small groups: 1*Patient, 1* physician , 1*nurse, 1*physiotherapist 1*camera man/woman
Competence of instructors	Expertise in clincial discipline<br>▪ Cognitive and practical clinical knowledge, experience in teaching skills, experience in student-oreinted teaching formats<br>Expertise in presentation media<br>▪ Experience in using modern audio/video media
Supervision	Instructors and tutors<br>▪ Intervention in learning situation is absolutely needed<br>▪ Intervention in learning situation upon request of participants
Media technique, presentation, video recording	User friendly technical equipment<br>▪ Projector, Laptop, loud speaker and Tablet-PC
Props per group	Props taken from clincial routine<br>▪ Patient bed, professional equipemnt (Stethoskope, blood presssure cuff, reflex hammer), Professional clothing<br>▪ Original aids from clinical routine (e.g. wheel chair, crutches)
Room planning	Coordination with officers in charge of all involved institutions: contact person of vocational school<br><br>*Class room capcity:*<br>▪ Large class room for work with whole group with presentation equipment<br>▪ Smaller class rooms with hospital equipment for video-recorded role plays
