# Supplementary material for: Recommendations to enhance constructivist-based learning in Interprofessional Education using video-based self-assessment
Source: GMS J Med Educ. 2016 Apr 29;33(2):Doc33. doi: 10.3205/zma001032 (PMC4895861; doi:10.3205/zma001032)
Supplement: Excerpt from the evaluation results [file JME-33-33-s-003.pdf]

How did the work with video-based self-assessment change your perception of your own working behavior?

I am more prone to search the communication with the physicians, since I understand better what is important for their work.
I did identiy some of my weaknesses and strengths. I know better, what might disturb my working routine and how I can adjust to this. I know better, what kind of flaws I did acquire due to a lack of self-control and how to improve my daily routine.
It was interesting to get to know, how the other health care professionals are working and it feels to do their job. I believe my understanding for the others did increase. I can better deal with the working profile of the other health care professions.
Quite a bit, since I could better perceive the effect of my own actions on others and how I do act and interact in different situations.
It will, especially in finding better arrangement with the other health care professions.
I guess, not that much, since the learning situation was rather artificial => not really applicablle in daily routine.
A lot, it can only be of advantage to observe one-selfs own actions and to identify the own errors by oneself.
One could reach a higher level of sensitization in respect to the selection of the own wording, orders, cooperation, and crisis management .
The perception of the own condition through others was better than the own perception (appearing more calm in the emergency situation than own perception); as physician higher need to take over responsibility in an emergency situation.
Despite the possibility to observe hidden reaction and actions, no change of my own perception of myself based on this scenario.
Even if I am not sure about the situation and what to do, I should stay calm to avoid feelings of insecurity in the team and for the patient.

What was the most prominent „moment of revelation“ for you during the seminar?

The contrast in the opinion of the advanced medical students and the senior physicians in respect to the delegation of tasks to other health care professional let me expect a more efficient distribution of tasks and a better interprofessional communication in the “next generation”.
My instruction for patient are not always very specific. In special situations (emergency) it is easy to forget even evident aspects of work, such as optimizing working condition with the patients by adjusting the height of the bed .
Insight in the work of other health care professionals, This opened my eyes and showed me, how to work in an interprofessional team. It was also very interesting to see the image of physiotherapy through the eyes of nurses and physicians.
See what other health care professionals are really doing and to reduce prejudices.
Cooperation and collaborative planning is important.
That, what I actually knew already, that the number of problems is lower, if people are talking with each other and not about each other.
To know what other health care professions are actually achieving and to realize their level of professionalism.
One realizes, how non-specific is the own wording in a given situation with a high level of assumption but without communicating details.
That, discussions occur on equal level rather easily when meeting the colleagues an other environment outside of daily routine., which is helpful for improving cooperation.
No „moment of revelation“.
Give orders precisely, avoid, terms like perhaps... or just a bit...

Which benefit did you draw from the workshop??

Exchange regarding rehabilitation in patients after stroke within and outside of the own profession.
Informal interesting discussions between participants and instructors in the breaks.
Extension of knowledge and sensitization regarding communication with other health care professions.
For me personally no substantial benefit, at least not yet, but may be later, as soon as my experiences increase.
It becomes easier to put oneself in the position of another profession and can , in the long run, optimize interprofessional cooperation.
Widen the own thinking, get an insight into the work of others.
Learn a new technique for positioning a patient from the physiotherapists.
It was interesting to get to know the point of view of the other health care professions, revise own habits.

Welchen Effekt hatte es für Sie, sich im Video zu sehen?

I was confirmed in my principal way of communicating, but I should work on my body language.
Self-control is possible in a completely unrestricted way, without being distorted in the memory.
Improvement of self reflexion in respect to verbal and non-verbal communication.
Perception of own verbal and non-verbal communication.
Observe and revise own errors.
Initially terrible feeling (dependent on role and working group).
Self assessment and identification and –revision of own errors
Self reflexion regarding the own behavior.
I appear more competent, than I thought and should act more self-confident.
In Video only once a patient and once as nurse. Role as physician could have possibly revealed unconscious reaction towards new situation.
Was surprised that I appeared more competent and self-confident than I thought and I felt during the role play and video recording .

Which effect did you realize upon assuming a different health care professional role?

To experience once a problem from a different angle and to see the patient from a different perspective.
It was funny and amusing to assume the role of a different health care professional.
Too see how helpless I was in the other healthcare profession and to see how much they accomplish.
Difficult, since the situation resembled strongly my routine field of work.
To know, what others are workingfor and what they achieve.
Feeling of insecurity when trying to adjust to the other profession.
Little new insight due to previous working experience in the field of nursing. Role of physiotherapist would have been more interesting for me.

How did your appreciation for the work of your colleagues change?

No big change in appreciation.
My appreciation for the other health care professions did increase. I got more understanding for errors in communication and interprofessional cooperation. I got a much better feeling for the relevance of the other professions and the need to work together towards the same goals.
Appreciation increased quite a lot. Understanding enables appreciation.
More understanding and more „sensitivity“ towards the other health care professions.
A bit more, since I can the other better within the context. No prejudices.
Not at all. Colleagues are colleagues.
I already appreciated my colleagues.
Towards the positive in respect to paying attention to each other.
Did increase.
I already had a high level of appreciation whaich was further confirmed.
No relevant change.
No obvious change, since I already tried to value the different health care professions equally.

How far can you put yourself now in the working position of your colleagues?

I have a much clearer idea regarding the tasks of the other health care professions.
I can judge much better now, where professional support is useful or disturbing. I know, how I can support the colleagues and support their work.
Through the knowledge and the self-experience I got a good insight into the work of the others. I can imagine much better, how and with which effort the other are working and what I can do to support them.
Better idea regarding the timing of tasks .
Difficult, time will show the effect.
Nice and precise insight, which facilitates understanding of the others.
Deepened my understanding
Not completely, but to a great deal
Insight did grow. I can perceive more and even non obvious aspects as before
The nursing staff carries more responsibilities than visisble in daily clinical routine.
No real change.

How did your attitude towards the other health care professions change?

Not at all, I am appreciating persons not professions..
I became more opened and less biased.
More empathy and understanding.
A bit, since I know better what they are doing. I can appreciate respect their work more.
I am respectful and friendly by nature and did not experience any changes.
Not at all (see 12.)
Towards the positive, especially in term of mutual consideration.
Increased appreciation.
My attitude was already positive before the workshop.
No real change.
No big change, since I always tried not to make a difference in my appreciation for the different health care professions.

What did you like most in this workshop?

The heterogenous but nevertheless very harmonic group, the discussion, also during the breaks.
The change of perspective and the video-based self-control are great tool and I enjoyed using them quite a lot.
The team was very nice and friendly and the class not too big. The informal discussion in the breaks were especially helpful.
Good balance between practical exercises and theory.
The video analysis and the chance to meet the other professional health care takers in an informal atmosphere.
Supervision, preparation, insight, self-reflexion.
The timing was very good and worked out exactly - not too little time and not too much time to get a deep insight in other fields and professions
Informal discussions with other health care professionals, video recording.
The exchange between the different groups of professions, especially the informal exchange during the breaks.
Playful introduction to foster self-perception and perception via the others during the role play. Chance to get in touch with the others during the breaks.
Stepwise increase in difficulty and complexity of the tasks.

What are your suggestions for improving the workshop?

Clear definition of tasks, introductory remarks regarding the over all of the workshop from the point of view of the funding organisation and regarding the potential consequences of the evaluation.
Clearer wording of the assigned tasks, keep the planned structure and responsibilities of the of instructor team (keep the allotted time slot , do not extend time slot at the cost of other discussion partners, let everybody finish his argument, let the moderator structure the discussion).
I would prefer when participants had a greater influence on the structure of the workshop. We would have had good ideas how to manage certain steps. The instructor did interfere too much.
Less detailed questions for video analysis. Limit discussion without trying to cover every detail.
Shorten the moderated video analysis discussion, reduce repetitions and redundance. Facilitate more interactions between students and less between. Shorten and simplify questionnaires.
Let discussion develop, do not interrupt discussions.
Provide more details for clinical case. Give more room to the students to express their thoughts.
Video analysis questionnaire are too complex, one can either watch the video or respond to the questions, but not both at the same time. Therefore please limit questionnaires..
Less theory and less redundant discussions, shorten workshop to one day or two half days.
I wish I would have had more unsupervised time for interprofessional exchange, possibly supported by providing a defined topic for the discussion.
Less guidance for the analysis would allow more exchange for impressions and perceptions from the students side, consider to reconstruct the clinical case towards a less compliant patient to become more realistic

Additional analysis of the questionaire:

What did you like most in the workshop?

- Appropiate number of participants, instructors and open discussions
- Good organisation, nice team
- Selected clinical case and video, insight in other health care professions, new experience when assuming another role Selbsterfahrung in einer anderen Rolle
- Informal discussion during the breaks
- Video-based self-reflexion, assuming other roles, reflecting own behavior, learning about the perception of others, video-records + subsequent discussion
- Opportunity of reflecting own behavior through own eyes and help of the others via the help of the video-record, getting insight in wotk of others in a easy and relaxed way, competent instructors and participants
- No negative judgement of errors, but searching for solutions of problems
- Exchange with other health care professionals and cooperative work to solve a clinical case
- Get to know the work of other health care professionals and reach a better understanding
- Possibility for professional exchange with other professions, nice and relaxed atmosphere helping to reach new awareness and get a good insight

What was missing in the workshop respectively what did you dislike?

- Fully trained health care professional should have the chance to participate in such a workshop, since errors are now realized after years of routine work.
- Enhance role description to improve role play
- Rules and instructions for reflexion
- Improve instructions and give more details
- Definition of tasks now always clear
- More information regarding long term goals of project
- Instruction for video assessment calling for too many details, too time consuming for the allotted time slot
- Moderator of discussion did not appreciate all arguments and comments adequately
- In my opinion the video analysis form was too complex for the allotted time
- Discussion was in part redundant, too many questions for the same topic
- Some details regarding patient treatment are very context dependent and require no information transfer in real patient handover
- Time frame too long, too many repetitions, effect could be achieved within a one day workshop
- Video analysis form too long and too complex, actually making the reflexion process more difficult
- Discussion part for the reflexion too long, could be shortened and more focused
