# Supplementary material for: Recommendations to enhance constructivist-based learning in Interprofessional Education using video-based self-assessment
Source: GMS J Med Educ. 2016 Apr 29;33(2):Doc33. doi: 10.3205/zma001032 (PMC4895861; doi:10.3205/zma001032)
Supplement: Evaluation tool [file JME-33-33-s-004.pdf]

		Opinion				Comment
		Yes	Mainly yes	Somehow yes	Mo	
1	**General**					
	The selected class rooms (size, location, noise, …)were very suitable.					
	Die technical equipment (media, technique, props) was very good.					
	The time frame for the workshop (date, day time, length) was appropriate.					
	The group size was appropriate.					
2	**Action**					
	I will be able to apply the newly acquired knowledge and skills.					
	The newly acquired knowledge and skills will be very useful in my working context .					
	Over all, this workshop increased my knowledge and skills substantially.					
3	**Interaction – Do you feel you are now empowered to …**					
	…perceive the need for mutual cooperation between the other health care professions more precisely?					
	…initiate adequate measures to change behaviour and attitude ?					
	…perceive your effect on others more profoundly?					
	…better understand the effect of others on your own perceptions?					
4	**Kommunication – Do you feel you are now empowered to …**	**Yes**	**Mainly yes**	**Soemhow yes**	**No**	**Comment**
	…identify the signs of good communication?					
	…identify signs of miscommunication?					
	…identify interprofessional conflict situations?					
	…Ihr eigenes Verhalten auf interprofessionelle Problemsituationen auszurichten?					

**5**	**Occupational knowledge**	**Very helpful**	**Helpful**	**Not so helpful**	**unneeded**	**Comment**	
	Was it helpful for you to get an insight into the working field of the other professions?						
**6**	**Video recording**						
	How did the work with video-based self control change your perception of your own working behavior?						
**7**	**Tasks**	**Way too low**	**Somehow too low**	**Appropiate**	**Somehow too high**	**Way too high**	**Comment**
	The level of difficulty of the assigned tasks was..						
	The support through instructors and tutors for completing the assigned tasks was …						
	The number of learning situations was …						
	For this workshop, my previous knowledge was						
**8**	What was your most prominent „moment of revelation“ in this interprofessional workshop?						
**9**	Which personal benefit did you experience in this interprofessional workshop?						
**10**	What was the effect of observing your own actions in the video records?						
	Rated on a scale from 1=no effect to 5= large effect?	① ② ③ ④ ⑤					
**11**	What effect did you experience by observing yourself assuming the role of a different health care giver?						
	Rated on a scale from 1=no effect to 5= large effect?	① ② ③ ④ ⑤					
**12**	How did your appreciation for the work of the other health care professionals change by getting a deeper insight into their job?						
**13**	Can you put yourself better in the position of your colleagues from other health care professions?						
**14**	How did your attitude towards the other health care professions change?						

		Very good	Good	OK	Not so good	Comment
15	How do you judge the instructional concept of video based self-assessment for teaching „Interprofessional cooperation“?					
16	My over all impression of the work shop is …					

17. What else would be of interest for the interaction with other health care professionals?

………

………

………

18. What did you like most in this workshop?

………

………

………

19. What are your suggestions for improving the workshop?

………

………

………
